# Temozolomide-induced biliary ductopenia: a case report

**DOI:** 10.1186/s13256-016-0804-z

**Published:** 2016-02-05

**Authors:** Asha Balakrishnan, Robert Ledford, Michael Jaglal

**Affiliations:** 1 Department of Internal Medicine, University of South Florida, Tampa, FL USA; 2 Department of Malignant Hematology, H. Lee Moffitt Cancer and Research Institute, 12902 Magnolia Drive, Tampa, FL 33612 USA

**Keywords:** Temozolamide, Glioblastoma, Hepatotoxicity, Biliary ductopenia, Vanishing bile duct syndrome

## Abstract

**Background:**

Temozolomide is an alkylating agent used along with concurrent radiation therapy in the treatment of glioblastoma. The primary adverse effect of temozolomide is bone marrow suppression with resulting cytopenias. There have been reported cases of temozolomide-induced hepatotoxicity, including fatal liver failure, associated with reactivation of the hepatitis virus or with concurrent use of other hepatotoxic drugs. In this report, we describe a unique mechanism of temozolomide-induced liver injury with supporting histopathology.

**Case presentation:**

Our patient, a 58-year-old African american woman with glioblastoma, was treated with concurrent radiation and temozolomide therapy. After 6 weeks of treatment, she developed worsening transaminitis and bilirubinemia with liver biopsy results consistent with drug-induced cholestasis and ductopenia. After cessation of drug treatment, her hyperbilirubinemia progressed with a peak bilirubin of 36.8 mg/dl. A repeat liver biopsy revealed severe biliary ductopenia consistent with vanishing bile duct syndrome.

**Conclusions:**

We present a rare case of a patient with biliary ductopenia as an adverse effect of temozolomide. During radiation and temozolomide therapy, blood counts and liver enzymes should be carefully monitored for the development of cholestatic liver injury. We recommend monitoring with weekly liver function tests and minimizing drugs that are metabolized by the liver during chemoradiation for glioblastoma.

## Background

Glioblastoma is the most common aggressive primary central nervous system tumor in adults. It accounts for 45.6 % of all malignant central nervous system tumors and has an incidence of 3.19 per 100,000 [1]. The current standard of care for the treatment of glioblastoma is surgical resection followed by adjuvant temozolomide and concurrent radiation therapy. The addition of temozolomide to radiotherapy significantly improves the median overall survival and progression-free survival compared with radiotherapy alone [2]. Temozolomide, an oral alkylating agent, enters the cerebrospinal fluid and does not require hepatic metabolism for activation [3]. The most common adverse effects of temozolomide include nausea, fatigue, and hematologic toxicities. In April 2014, Merck Canada released an announcement acknowledging the association of temozolomide use with liver injury, including fatal hepatic failure, which may present up to 16 weeks following drug initiation [4]. Various mechanisms of hepatotoxicity have been described in the literature, including the reactivation of hepatitis virus, the use of concomitant hepatotoxic medications, and cholestatic hepatitis [5–11]. We present a case of vanishing bile duct syndrome secondary to temozolomide use in the treatment of a patient with glioblastoma. To the best of our knowledge, we report only the second such case in the literature [12].

## Case presentation

A 58-year-old previously healthy Caucasian woman initially presented to our institute with headaches, nausea, and photophobia. She was found to have a 4.7 × 4.2 × 3.6–cm hemorrhagic right temporal lobe mass. She underwent emergent right craniotomy and mass resection, and the tumor pathology was consistent with glioblastoma. In laboratory examinations to evaluate her baseline hepatic function before chemoradiation, the results were aspartate transaminase 22 IU/L (normal range 5–34 IU/L), alanine transaminase 14 IU/L (normal range 5–55 IU/L), alkaline phosphatase 90 U/L (normal range 40–150 U/L), and total bilirubin 0.4 mg/dl (normal range 0.2–1.2 mg/dl). The patient’s medications during chemoradiation were temozolomide, trimethoprim-sulfamethoxazole, and ondansetron, and no supplements (herbal or over-the-counter vitamins) were used during chemoradiation.

Six weeks after initiation of temozolomide and radiation therapy, the patient was admitted to the hospital for rapidly increasing transaminitis (aspartate transaminase 425 IU/L, alanine transaminase 1028 IU/L), elevated alkaline phosphatase (291 U/L), and hyperbilirubinemia (total bilirubin 7.9 mg/dl) discovered in routine weekly blood work. She presented with jaundice and dark urine but otherwise was asymptomatic. The results of her infection workup (hepatitis panel, cytomegalovirus, herpes simplex virus, Epstein-Barr virus, HIV) and her autoimmune workup (antinuclear antibody, smooth muscle antibody, antimitochondrial antibody, immunoglobulins) were negative, and her liver ultrasound was unremarkable. She was not on any other hepatotoxic medications at the time of admission. Temozolomide was held on admission, and, although her aspartate transaminase and alanine transaminase levels improved, her total bilirubin continued to rise. Her initial liver biopsy pathology was consistent with a cholestatic pattern of injury with mild ductopenia and portal eosinophilia consistent with a drug-induced liver injury.

Three weeks after admission, the patient’s total bilirubin rose to a peak of 36.8 mg/dl, and repeat liver biopsy revealed progression to severe biliary ductopenia, consistent with vanishing bile duct syndrome. She was started on a trial of ursodeoxycholic acid and zinc with gradual improvement of her transaminitis and hyperbilirubinemia over several months. After discontinuation of temozolomide, she completed radiation. She did not have any further therapy except radiation while her hepatic function normalized. She relapsed approximately 9 months later and was then treated with re-resection followed by reradiation. Since her reradiation, the patient has had no evidence of recurrence and undergoes follow-up magnetic resonance imaging every 3 months. She has been followed for a total of 2 years and is currently without evidence of disease.

## Discussion

The association of liver injury with the use of temozolomide has become more established in recent years. The mechanism of temozolomide-induced hepatotoxicity is unclear, as the drug does not require hepatic metabolism for activation [3]. Vanishing bile duct syndrome is a rare manifestation of temozolomide-induced liver injury. In a recent case report, Mason *et al.* [12] described a 62-year-old Indian woman who developed signs of liver failure 17 days after initiation of temozolomide therapy, with a peak bilirubin of 289 mg/dl. A liver biopsy done at day 30 confirmed vanishing bile duct syndrome, and she was treated with ursodeoxycholic acid and cholestyramine with resolution of liver enzymes by day 129. Several triggers of vanishing bile duct syndrome have been described, including autoimmune disorders, infections, congenital diseases, malignancy, and medications [13]. Treatment of vanishing bile duct syndrome varies with the underlying cause, but strategies generally include supportive care, withdrawal of the offending medication, treatment of the underlying infection, ursodeoxycholic acid, and immunosuppressive agents.

## Conclusions

Biliary ductopenia is a rare manifestation of temozolomide-induced liver injury. Blood counts and liver enzymes should be monitored closely during concurrent temozolomide and radiation therapy for glioblastoma. We recommend monitoring with weekly liver function tests and minimizing drugs that are metabolized by the liver during chemoradiation for glioblastoma.

## Consent

Written informed consent was obtained from the patient for publication of this case report and any accompanying images. A copy of the written consent is available for review by the Editor-in-Chief of this journal.

## References

[1] Ostrom QT, Gittleman H, Liao P, Rouse C, Chen Y, Dowling J, et al. CBTRUS statistical report: primary brain and central nervous system tumors diagnosed in the United States in 2007-2011. Neuro Oncol. 2014;16 Suppl 4:iv1–63. 10.1093/neuonc/nou223 PMC4193675 25304271

[2] Stupp R, Mason WP, van den Bent MJ, Weller M, Fisher B, Taphoorn MJ, et al. Radiotherapy plus concomitant and adjuvant temozolomide for glioblastoma. N Engl J Med. 2005;352(10):987–96. 10.1056/NEJMoa043330 15758009

[3] Friedman HS, Kerby T, Calvert H. Temozolomide and treatment of malignant glioma. Clin Cancer Res. 2000;6(7):2585–97. 10914698

[4] Merck Canada. Health Canada endorsed important safety information on TEMODAL® (temozolomide): association of TEMODAL® (temozolomide) with the risk of hepatic injury [news release]. 7 May 2014; http://www.merck.ca/Assets/News/TEMODAL%20HPC%20ENGLISH%20-%20FINAL.pdf. Accessed 15 January 2016.

[5] Becker F, Hecht M, Schmidtner J, Semrau S, Fietkau R. Temozolomide-induced liver damage: a case report. Strahlenther Onkol. 2014;190(4):408–10. 10.1007/s00066-013-0519-7 24452817

[6] Chheda MG, Drappatz J, Greenberger NJ, Kesari S, Weiss SE, Gigas DC, et al. Hepatitis B reactivation during glioblastoma treatment with temozolomide: a cautionary note. Neurology. 2007;68(12):955–6. 10.1212/01.wnl.0000259430.48835.b5 17372135

[7] Sarganas G, Orzechowski HD, Klimpel A, Thomae M, Kauffmann W, Herbst H, et al. Severe sustained cholestatic hepatitis following temozolomide in a patient with glioblastoma multiforme: case study and review of data from the FDA adverse event reporting system. Neuro Oncol. 2012;14(5):541–6. 10.1093/neuonc/nos056 PMC3337307 22394496

[8] Ohno M, Narita Y, Miyakita Y, Ueno H, Kayama T, Shibui S. Reactivation of hepatitis B virus after glioblastoma treatment with temozolomide: case report. Neurol Med Chir (Tokyo). 2011;51(10):728–31. 10.2176/nmc.51.728 22027252

[9] Niewald M, Berdel C, Fleckenstein J, Licht N, Ketter R, Rübe C. Toxicity after radiochemotherapy for glioblastoma using temozolomide - a retrospective evaluation. Radiat Oncol. 2011;6:141. 10.1186/1748-717X-6-141 PMC3213071 22017800

[10] Grewal J, Dellinger CA, Yung WK. Fatal reactivation of hepatitis B with temozolomide. N Engl J Med. 2007;356(15):1591–2. 10.1056/NEJMc063696 17429098

[11] Dixit S, Hingorani M, Afzal P, Campbell AP. Temozolomide induced liver injury. Acta Neurol Belg. 2011;111(3):249–51. 22141295

[12] Mason M, Adeyi O, Fung S, Millar BA. Vanishing bile duct syndrome in the context of concurrent temozolomide for glioblastoma. BMJ Case Rep. 2014. doi: 10.1136/bcr-2014-208117 PMC4248108 25432915

[13] Reau NS, Jensen DM. Vanishing bile duct syndrome. Clin Liver Dis. 2008;12(1):203–17. 10.1016/j.cld.2007.11.007 18242505

